# Editorial: Environmental risk factors in noncommunicable diseases: new insights into the molecular mechanisms

**DOI:** 10.3389/fpubh.2025.1632200

**Published:** 2025-07-15

**Authors:** Alica Pizent, Andres Cardenas, Lidia Mínguez-Alarcón

**Affiliations:** ^1^Division of Occupational and Environmental Health, Institute for Medical Research and Occupational Health, Zagreb, Croatia; ^2^Department of Epidemiology and Population Health, Stanford University School of Medicine, Stanford, CA, United States; ^3^Department of Medicine, Brigham and Women's Hospital and Harvard Medical School, Boston, MA, United States; ^4^Department of Environmental Health, Harvard T. H. Chan School of Public Health, Boston, MA, United States

**Keywords:** air pollution, bisphenols, oxidative stress, inflammatory signaling, metabolic dysfunction, thrombotic activation

Non-communicable diseases (NCDs), including cardiovascular disease, metabolic syndrome, and obesity, represent a growing global health challenge, with environmental risk factors now recognized as key contributors to their development and progression. Air pollution, particulate matter (PM), and endocrine-disrupting chemicals (EDCs) can modify genetic risk and alter epigenetic variation, impair cellular homeostasis, and accelerate the onset of chronic diseases. As exposure to these pollutants rises globally, understanding the molecular mechanisms linking environmental stressors to NCDs has become a public health and research priority. Recent advances in molecular epidemiology and exposome research along with new methodologies could advance our understanding of the health impact of various environmental factors. This Research Topic brings together studies that integrate epidemiological and mechanistic insights, shedding new light on how environmental pollutants reprogram biological systems and identifies novel targets for prevention and intervention to reduce the burden of NCDs. The manuscripts featured in this Research Topic explore a range of environmental factors, their molecular impacts, and their contributions to various NCDs.

The Jiangsu study in China analyzed 1,417,773 cardiovascular deaths over seven years and found that acute exposure to PM significantly increased mortality risks. Zhu et al. reported non-linear relationships between short-term exposure to PM_2.5_ and PM_10_ and daily total mortality from cardiovascular diseases. While districts with lower average PM levels showed greater relative mortality increases per 10 μg/m^3^ PM rise, high-exposure regions had the highest absolute mortality burden due to sustained pollution. The effects were more pronounced in females, older adults, and regions with lower baseline PM levels. The authors hypothesized that short-term PM exposure triggers inflammation via free radicals, increasing blood coagulation and plasma viscosity, thereby heightening the risk of ischemic stroke. The study's non-linear exposure-response curves highlight that even a modest increase in pollution levels pose serious health risks, underscoring the need for stricter air quality regulations in high-exposure regions.

Similarly, Hantrakool et al. identified PM_2.5_ and PM_10_ as critical drivers of cardiovascular disease and used lagged exposure models to capture delayed effects. The Tai prospective study assessed hemostatic changes in 30 healthy males exposed to seasonal PM fluctuations. Short-term ambient PM exposure shortened prothrombin time, induced platelet activation and enhanced coagulation, elevating risk of stroke and myocardial infarction.

In the cross-sectional study of the relationship between long-term air pollution and multimorbidity (≥2 coexisting chronic conditions) in 364,144 UK Biobank participants, Ronaldson et al. demonstrated association of exposure to ambient PM_2.5_ and nitrogen dioxide (NO_2_) with increased risk and severity of multimorbidity, particularly involving respiratory, cardiovascular, and neurological disease clusters. These findings position air pollution as a key modifiable environmental risk factor for non-communicable diseases, acting through molecular mechanisms such as oxidative stress, systemic inflammation, and immune activation. The study supports the view that chronic exposure to air pollutants may accelerate multi-organ damage and highlights the importance of air quality interventions to mitigate the burden of multimorbidity in the population.

Yu et al. investigated the direct and indirect effects of air pollution on overweight and obesity using nationally representative data from the China Family Panel Survey. The obesogenic effects of PM_2.5_ and PM_10_ were more pronounced in men, adults older than 40 years, and rural populations compared to women, younger individuals, and urban residents. Employing regression discontinuity and structural equation modeling, the authors showed pollutant-specific effects on obesity-related outcomes, mediated by social behavior determinants, such as physical activity and sedentary behavior, sleep quality, smoking, alcohol consumption, and mental health. The authors suggested that changes in lifestyle and behavior may contribute to mitigate pollution-induced metabolic disruptions in high-risk populations.

Finally, the issue includes an intriguing study by Xiao et al. that used advanced imaging techniques to examine how exposure to bisphenol analogs affects glucose metabolism. In a retrospective analysis performed in 20 male and 20 female adults, the study showed that exposure to bisphenol A (BPA) and bisphenol S (BPS) is associated with distinct, sex- and organ-specific alterations in tissue glucose metabolism as measured by 18F-fluorodeoxyglucose positron emission tomography/computed tomography (FDG PET/CT) imaging. In males, higher urinary BPA was associated with increased hepatic glucose uptake, suggesting that BPA may promote metabolic dysfunction through mitochondrial impairment and enhanced glycolysis. BPS in males was associated with reduced thyroid activity, contributing to disruption of thyroid hormone synthesis and hypothyroidism. In females, BPS exposure was associated with increased cerebral cortex glucose metabolism, possibly reflecting neuroinflammatory or hormonal effects. These findings offer new insights into the molecular pathways by which these EDCs contribute to metabolic diseases.

Taken together, the studies presented in this Research Topic significantly enhances our understanding of how environmental factors drive the pathogenesis of NCDs. Yet, important questions remain regarding the long-term health impact of environmental exposures, their cumulative effects, and the specific molecular pathways involved. While the presented studies mostly evaluated PM in relation to NCDs, research on other important air pollution biomarkers warrant further evaluation. In addition, exposure to traditional EDCs is decreasing (e.g., BPA) whereas exposure to new replacements is on rise (not only BPS but also BPF). Thus, newer study cohorts should explore health effects of exposure to these EDCs and other emerging contaminants that were introduced in the market without proving their safety in humans. Lastly, studies among racialized communities are critical to better understand how exposure to environmental pollutants affect NCDs in the most vulnerable populations. Continued research into these areas is essential for identifying effective prevention strategies and informing policy decisions aimed at mitigating the health risk associated with environmental pollution.

We would like to thank all authors for their significant contributions and the reviewers for their invaluable feedback in shaping this Research Topic. We hope that this Research Topic provides a foundation for future studies on this topic.

